# Interference Structures in the High-Order Above-Threshold Ionization Spectra of Polyatomic Molecules in a Bicircular Laser Field

**DOI:** 10.3390/molecules30142946

**Published:** 2025-07-11

**Authors:** Elvedin Hasović, Azra Gazibegović-Busuladžić, Mustafa Busuladžić

**Affiliations:** 1 Faculty of Science, University of Sarajevo, 71000 Sarajevo, Bosnia and Herzegovina; elvedin.hasovic@pmf.unsa.ba (E.H.); azragb@pmf.unsa.ba (A.G.-B.); 2 Faculty of Medicine, University of Sarajevo, 71000 Sarajevo, Bosnia and Herzegovina

**Keywords:** high-order above-threshold ionization, polyatomic molecules, molecular spectra, bicircular laser field, interference minima, molecular structure

## Abstract

We analyze the high-order above-threshold ionization (HATI) process of a small polyatomic molecule with C_3_ symmetry, which is induced by a bicircular strong laser field. This field consists of two coplanar, counter-rotating, circularly polarized components with frequencies rω and sω where *r* and *s* are integers. In our study, we use an improved molecular strong-field approximation to obtain electron energy-angle-resolved and momentum spectra of the BF_3_ molecule. We analyze the contributions of individual atoms as well as the impact of molecular symmetries on these spectra. We find that these spectra are significantly affected by the characteristics of the molecule and the laser-field parameters. Furthermore, we observe pronounced interference minima in the HATI spectra. We demonstrate that these minima result from the destructive interference of rescattered wave packets from different atomic centers, and we determine the conditions under which they occur, including two-, three-, and four-center interference.

## 1. Introduction

In recent decades, interest in researching the interaction of strong laser fields with matter has increased significantly [1,2]. These interactions can lead to various atomic or molecular processes, which can be categorized into two groups: processes that occur only in the presence of a laser field and processes that can also take place without it. This paper focuses on the former, known as laser-induced processes [3,4]. Examples of these processes include high-order harmonic generation (HHG) and high-order above-threshold ionization (HATI). Both can be explained by the so-called three-step model. The initial step in these processes is above-threshold ionization (ATI), which Pierre Agostini and colleagues first observed experimentally 45 years ago [5]. In the ATI process, a quantum-mechanical system, such as an atom or molecule, can absorb more photons during its interaction with a strong laser field than necessary for ionization. The emitted electrons are then accelerated by the alternating electric field in the second step. If they do not interact further with the parent atomic or molecular ions, they are referred to as direct or ATI electrons. However, in the third step, the emitted electrons may experience additional interactions with the parent ion. More precisely, a returning electron may elastically scatter off its parent ion before moving toward the detector. During this process, which is called high-order ATI (HATI) [6,7], the electron can absorb many more photons from the laser field than during the direct ATI process. The energy spectrum of the HATI process is characterized by a long plateau that ends abruptly. In addition to rescattering, the returning electron can recombine with the parent ion and emit the excess energy in the form of a high-energy photon. This process is known as high-order harmonic generation (HHG). Discovered in two laboratories 40 years ago [8,9], HHG is a highly nonlinear process. One of the most significant applications of HHG is the generation of attosecond pulses, which enables the investigation of electron dynamics on the atomic scale [10,11,12]. Specifically, bright, phase-matched circularly polarized extreme ultraviolet high-harmonic radiation was obtained using a bichromatic counter-rotating circularly polarized laser field (i.e., bicircular field) [13].

In this paper, we are interested in the HATI process on polyatomic molecules governed by a complex laser field. While the (H)ATI process of diatomic and similar small molecules has been investigated in numerous papers, direct ATI and high-energy HATI of more complex, polyatomic molecules have been examined in fewer cases so far. In reference [14], the authors theoretically investigated direct above-threshold ionization (ATI) of ethylene, benzene, and fluorobenzene molecules in a strong, linearly polarized laser field. They demonstrated that the orientation dependence of ionization yields is primarily determined by the nodal surface structure of the molecular orbitals. Kumarappan et al. examined the angular distribution of photoelectrons ejected from carbon sulfide molecules in response to a linearly polarized field, while using short nonionizing laser pulses to control alignment of gas phase molecules. Experiments showed that molecule ionization is significantly dependent on the angle between the polarizations of the aligning and ionizing laser fields [15]. Strong-field processes of various polyatomic molecules were studied in references [16,17], with a focus on the low-energy features of the photoelectron spectrum. Zhou et al. investigated the interference structure in ATI spectra of the polyatomic molecule SF_6_ using IR + XUV two-color laser fields [18]. A smaller number of papers have been devoted to analyzing rescattered or HATI electrons on polyatomic molecules. Laser-induced electron diffraction (LIED), which is based on the HATI process, has been utilized to extract information about molecular targets [19,20]. Findings on LIED in polyatomic molecules such as C_2_H_2_, OCS, CS_2_, including theoretical approaches and experimental data, can be found in the recently published review article and references therein [21]. In most of the above-mentioned papers, a linearly polarized field was considered.

Strong-field ionization in two-color circularly polarized laser fields was first experimentally investigated for atomic targets. Among the first such experiments was the investigation of electron–ion rescattering for argon in bicircular fields, studied both experimentally and through theoretical approaches [22]. Recently, Beaulieu et al. investigated the strong-field ionization of polyatomic molecules in a bicircular laser field and found that the observed interference of chiral molecules exhibits asymmetry along the direction of light propagation and increases the sensitivity of the attoclock scheme to the chirality of the molecules [23]. Experimental observations of strong-field ionization of S- and R-propylene oxide in circularly polarized two-color laser fields compared to heuristic model based on electrons in chiral initial states are presented in [24].

Various theories have been developed to analyze the (H)ATI process. One way to theoretically describe the interaction of strong laser fields with atoms and molecules is to solve the corresponding time-dependent Schrödinger equation (TDSE). An analytical solution is not possible even for the simplest atomic targets, and numerical solutions of the TDSE are very demanding and time-consuming, especially for molecular targets [25,26,27,28]. Additionally, there are theories that use analytical approaches and employ various approximations. One such theory, which has been successfully used for decades to describe the interaction of strong laser fields with matter, is the strong-field approximation (SFA). Within the SFA, the interaction between the emitted electron and the residual ion is neglected during electron propagation in the laser field. The electron continuum state is then determined solely by its interaction with the applied laser field. SFA results are often analyzed alongside TDSE as they enable physical insight into the ionization process, especially concerning quantum interference and instants of subsequent parts of the process. A molecular SFA (MSFA) was derived to describe direct ATI [29,30], while the so-called improved MSFA (IMSFA) was developed to analyze the HATI process of molecules exposed to strong laser fields [31,32,33,34]. It is important to emphasize that IMSFA was used to successfully simulate experimental HATI results on small molecules [35,36].

This article is organized as follows: In Section 2, we provide the necessary theoretical background by briefly discussing the IMSFA, the bicircular laser field, and the geometry of the process under consideration. In Section 3, we present our numerical results for the BF_3_ molecule, as a representative of molecules with C_3_ symmetry, focusing on the destructive interference patterns observed in the corresponding HATI spectra. Finally, in Section 4, we present our conclusions. Unless otherwise stated, we use atomic units throughout the article ($\hbar =e=m=4\pi \varepsilon_{0}=1$).

## 2. Theory

We consider a polyatomic molecule as a system of *N* atomic (ionic) centers and an electron that can be released under the influence of the laser field with the electric field vector E(t) and vector potential A(t). We describe the interaction of the molecule with the laser pulse in dipole approximation and length gauge. We apply the *S*-matrix theory to the molecular ATI and HATI processes and follow the same theoretical approach which was developed before [29,30,33]. The observable quantity is the differential ionization rate, defined as

(1) $$w_{\mathrm{fi}}\left(n\right)=2\pi p_{f}{\left|T_{\mathrm{fi}}\left(n\right)\right|}^{2},$$

where the *T*-matrix element for ionization with absorption of *n* photons from the laser field is

(2) $$T_{\mathrm{fi}}\left(n\right)=\int_{0}^{T}\frac{dt}{T}\left[F_{\mathrm{fi}}^{\left(0\right)}\left(t\right)+F_{\mathrm{fi}}^{\left(1\right)}\left(t\right)\right]e^{i[U_{f}\left(t\right)+n\omega t]},$$

with $U_{f}\left(t\right)=p_{f}\cdot \alpha \left(t\right)+\int^{t}d\tau A^{2}\left(\tau \right)/2-U_{p}t$, where $p_{f}$ is the final-momentum vector, $U_{p}$ is the ponderomotive energy defined as $U_{p}=\int_{0}^{T}dtA^{2}\left(t\right)/\left(2T\right)$, and $\alpha \left(t\right)=\int^{t}d\tau A\left(\tau \right)$. The *T*-matrix element is the sum of two contributions: the first term $F_{\mathrm{fi}}^{\left(0\right)}\left(t\right)$ describes the direct electrons, while $F_{\mathrm{fi}}^{\left(1\right)}\left(t\right)$ describes the rescattered electrons, which are responsible for the high-energy plateau in HATI spectra. For neutral polyatomic molecules, the direct-electron term, calculated with the IMSFA, is [30]

(3) $$\begin{array}{ccc} F_{\mathrm{fi}}^{\left(0\right)}\left(t\right) & = & \sum_{j=1}^{N}e^{-ip_{f}\cdot \rho_{j}}\sum_{a}c_{ja}\langle p_{f}+A\left(t\right)|E\left(t\right)\cdot r|\psi_{a}\rangle , \end{array}$$

where $\rho_{j}$ is defined by Equation (24) in [30] while $c_{ja}$ and $|\psi_{a}\rangle$ determines the ground-state electronic function in a molecule.

The corresponding matrix-element that describes rescattered electrons can be written in the following form [33]:

(4) $$\begin{array}{ccc} & & F_{\mathrm{fi}}^{\left(1\right)}\left(t\right)=-ie^{-iS_{k_{\mathrm{st}}}\left(t\right)}\int_{0}^{\infty }d\tau {\left(\frac{2\pi }{i\tau }\right)}^{3/2}e^{i\left[S_{k_{\mathrm{st}}}\left(t^{\prime }\right)-\Delta E\left(\left\{R_{0}\right\}\right)\tau \right]}\\ & & \times \sum_{j=1}^{N}e^{iK_{\mathrm{st}}\cdot \rho_{j}\left(\left\{R_{0}\right\}\right)}V_{eK_{\mathrm{st}}}^{j}\sum_{l=1}^{N}e^{-ik_{\mathrm{st}}\cdot \rho_{l}}\sum_{a}c_{la}\langle k_{\mathrm{st}}+A\left(t^{\prime }\right)|E\left(t^{\prime }\right)\cdot r|\psi_{a}\rangle , \end{array}$$

with $t^{\prime }=t-\tau$, $K_{\mathrm{st}}=k_{\mathrm{st}}-p_{f}$, $k_{\mathrm{st}}=-\int_{t^{\prime }}^{t}dt^{\prime \prime }A\left(t^{\prime \prime }\right)/\tau$ the stationary electron momentum, $S_{k}\left(t\right)=\int^{t}dt^{\prime }{\left[k+A\left(t^{\prime }\right)\right]}^{2}/2$, and $V_{eK_{\mathrm{st}}}^{j}$ is the Fourier transform of the rescattering potential on the *j*-center. We assume that the ionization process occurs from the highest occupied molecular orbital (HOMO) of the molecule, which is written as a linear combination of atomic orbitals [30]. The appendix in [30] provides detailed information about the calculation of ionization matrix elements for a Gaussian basis set.

A bicircular laser field is a superposition of two coplanar counter-rotating fields having the angular frequencies rω and sω, which are integer multiples of the same fundamental frequency ω=2π/T. It is defined by

(5) $$\begin{array}{c} E\left(t\right)=\frac{i}{2}\left(E_{1}{\hat{e}}_{+}e^{-ir\omega t}+E_{2}{\hat{e}}_{-}e^{-is\omega t}\right)+c.c., \end{array}$$

where ${\hat{e}}_{\pm }=\left({\hat{e}}_{x}\pm i{\hat{e}}_{y}\right)/\sqrt{2}$, with ${\hat{e}}_{x}$ and ${\hat{e}}_{y}$ the real unit polarization vectors along the *x* and *y* axes, respectively. In (5), $E_{j}$ and $I_{j}=E_{j}^{2}$ are the electric-field vector amplitude and the intensity of the *j*th field component with the helicities $h_{j}$ ($h_{1}=1$, $h_{2}=-1$), respectively. Introducing arbitrary phases $\phi_{1}$ and $\phi_{2}$ in the definition of the bicircular field, the field components can be written as [37]

(6) $$\begin{array}{ccc} E_{x}\left(t\right) & = & \left[E_{1}\sin\left(r\omega t+\phi_{1}\right)+E_{2}\sin\left(s\omega t+\phi_{2}\right)\right]/\sqrt{2},\\ E_{y}\left(t\right) & = & \left[-E_{1}\cos\left(r\omega t+\phi_{1}\right)+E_{2}\cos\left(s\omega t+\phi_{2}\right)\right]/\sqrt{2}. \end{array}$$

**Figure 1:**
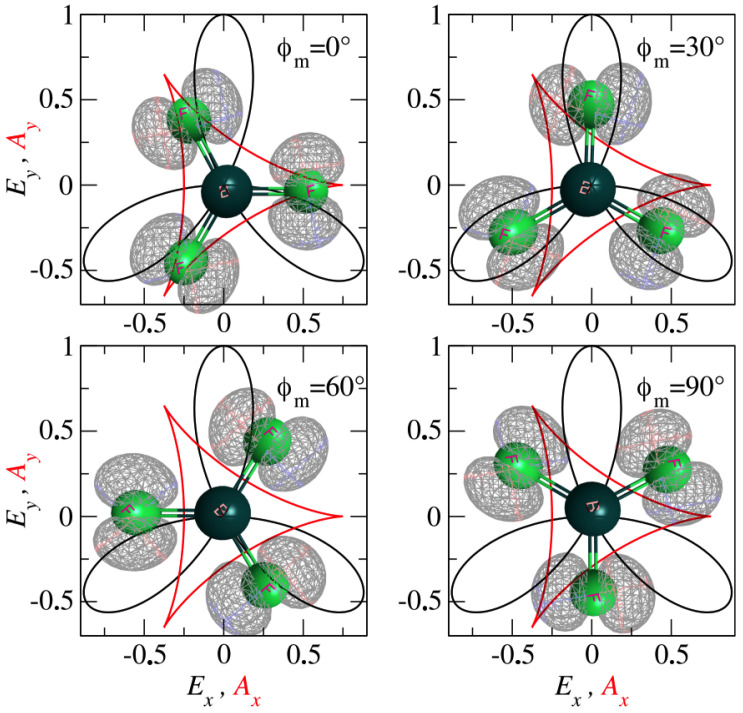
Polar diagram of the electric field vector E(t), 0≤t≤T (black lines) and the corresponding vector potential A(t) (red lines) of the ω–2ω bicircular fields with equal intensities of the field components. The equilibrium geometry of the BF_3_ planar molecule and its corresponding HOMO orbital is presented for four different orientations of the molecular plane with respect to the polarization plane of the laser field: $\phi_{m}=0^{{}^{\circ}}$ (**upper left panel**), $\phi_{m}=30^{{}^{\circ}}$ (**upper right panel**), $\phi_{m}=60^{{}^{\circ}}$ (**bottom left panel**), $\phi_{m}=90^{{}^{\circ}}$ (**bottom right panel**). The central atom in the molecule is boron (dark green circle), while the remaining three are fluorine (bright green circle) atoms.

In this paper, we assume equal component strengths and fix the relative phases to zero ($E_{1}=E_{2}=E_{L}$, $\phi_{1}=\phi_{2}=0$). In Figure 1, we plot the electric-field vector E(t), 0≤t≤T (black lines) and the corresponding vector potential A(t) (red lines) of the ω−2ω bicircular field. It is obvious that both the electric field and the vector potential obey a $120^{{}^{\circ}}$ rotational symmetry, which is convenient when probing a molecule with the same rotational symmetry.

## 3. Results and Discussion

In this section, we will present our numerical results. We examine the high-order above-threshold ionization (HATI) process of BF_3_ molecule under the influence of a ω–2ω bicircular laser field as defined in Equation (6). The calculation of molecular orbitals was performed using the GAMESS quantum chemistry package with the cc-pVTZ basis set [38,39,40]. The HOMO of the BF_3_ molecule, which has a calculated ionization potential ($I_{p}$) of 17.99 eV and $A_{2}$ symmetry is presented in Figure 1. It is important to note that for the BF_3_ molecule, two low-lying orbitals are energetically close to the HOMO. These orbitals are antisymmetric with respect to the reflection about the horizontal xy plane, resulting in a corresponding ionization matrix element of zero. Thus, it is reasonable to analyze the ionization process solely from the HOMO. The equilibrium geometry, along with the orbital energies and symmetries, can be found in two referenced sources [39,40]. In all calculations, the intensities of the bicircular laser-field components are set to $1\cdot 10^{14}$ W/cm^2^, with a fundamental wavelength of 800 nm. Here the wavelength of the laser is much larger than the considered molecule, and intensity of the bicircular laser field is not very high, so we used a dipole approximation as it simplifies calculations, treating electric field as constant across the molecule and neglecting the influence of magnetic field.

**Figure 2:**
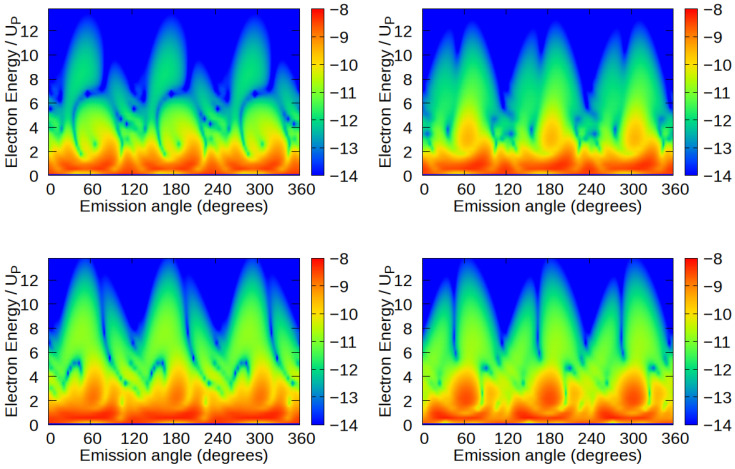
Logarithm of the differential ionization probability (HATI spectra) of the BF_3_ molecule as a function of the electron kinetic energy and emission angle. All contributions are taken into account, i.e., total HATI spectra are presented. The intensity of both components of the bicircular ω–2ω laser field is $10^{14}$ W/cm^2^, with a fundamental wavelength of 800 nm. The molecular plane corresponds to the polarization plane of the applied laser field while the molecule is rotated by angles of $\phi_{m}=0^{{}^{\circ}}$ (**upper left panel**), $\phi_{m}=30^{{}^{\circ}}$ (**upper right panel**), $\phi_{m}=60^{{}^{\circ}}$ (**bottom left panel**), and $\phi_{m}=90^{{}^{\circ}}$ (**bottom right panel**) with respect to the laser-field polarization vector.

We now present the HATI spectra of the BF_3_ molecule in an ω–2ω field for various molecular orientations. We consider the case where the molecular plane corresponds to the laser-field polarization plane. We rotate the molecule by the following angles $\phi_{m}=0^{{}^{\circ}},30^{{}^{\circ}},60^{{}^{\circ}},$ and $90^{{}^{\circ}}$ (Figure 1) and calculate the angle and energy distributions of the emitted HATI electrons. The corresponding electron distributions are shown in Figure 2 for the previously mentioned molecular orientations. In all four cases, the spectra consist of three broad peaks at emission angles of approximately $\theta \approx 60^{{}^{\circ}},180^{{}^{\circ}}$ and $300^{{}^{\circ}}$. These directions are opposite to the direction of the vector potential at the ionization time (i.e., peaked values of the vector potential) and the electrons are accelerated in these directions to their maximum energies.

An interesting feature of the presented spectra is the rotational symmetry by an angle of $120^{{}^{\circ}}$. The bicircular field E(t) and the vector potential A(t) obey the following dynamical symmetry: Rotation by the angle $\alpha_{j}=-2\pi jr/\left(r+s\right)$ about the *z* axis is equivalent to a translation in time by $\tau_{j}=jT/\left(r+s\right)$, i.e.,

(7) $$R_{z}\left(\alpha_{j}\right)E\left(t\right)=E\left(t+\tau_{j}\right),$$

where *j* is an integer. It has been demonstrated that the spectra of photons generated in HHG, as well as the spectra of (H)ATI electrons, obtained by placing atoms in a bicircular field, exhibit the same type of the symmetry [34,41,42,43]. Furthermore, it was shown that placing an arbitrary molecule in a bicircular laser field breaks this symmetry (see the review article [44] and references therein). However, placing a molecule that is rotationally symmetric by $120^{{}^{\circ}}$, such as the BF_3_ molecule, in an ω–2ω field causes the HATI spectra to obey the same symmetry as the laser field and the molecule, which is evident from our results. Even taking into account non-dipole effects should not break this symmetry, as we analyze photoelectron spectra in the plane perpendicular to the light propagation direction, and non-dipole effect introduces an asymmetry in the photoelectron momentum distribution along the direction of light propagation [45,46].

Another interesting feature of the presented spectra of the BF_3_ molecule is the appearance of deep minima in the high-energy part of the spectrum for specific electron emission angles for each molecular orientation considered. Similar minima were discovered earlier for diatomic molecules and were explained as the destructive interference of rescattering wave packets spreading from different atomic centers [31,35,47,48,49]. Because molecules are multi-center systems, ionization and rescattering can occur at different centers, creating interference patterns in the electron spectrum. Our theoretical model allows us to examine this phenomenon more closely. Since the transition matrix element is expressed as the sum of contributions of ionization as well as rescattering matrix elements from different atomic centers, we can calculate the so-called partial contributions, i.e., the contributions to the ionization and rescattering of each atom in the molecule.

**Figure 3:**
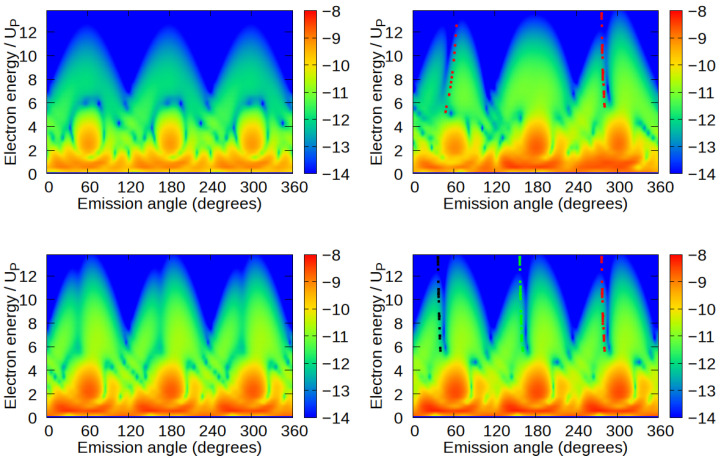
HATI spectra obtained by taking into account partial contributions of rescattering matrix element for boron atom only (**upper left panel**), two fluorine atoms (**upper right panel**), three fluorine atoms (**lower left panel**), and all atoms (**lower right panel**) for the same laser-field parameters as in Figure 2 and $\phi_{m}=90^{{}^{\circ}}$. Gradual inclusion of atomic centers in calculations demonstrates multi-center interference minima. Dotted and dashed lines mark the calculated approximate position of two-center minima.

In Figure 3, we show HATI spectra obtained by taking into account partial contributions of rescattering matrix element for boron atom only (upper left panel), two fluorine atoms (upper right panel), three fluorine atoms (lower left panel), and all atoms (lower right panel) for the same laser-field parameters as in Figure 2 and $\phi_{m}=90^{{}^{\circ}}$. When rescattering from only one atomic center is taken into account, the minima in the high-energy part of the spectra do not appear. The absence of these minima can be considered as the first indication that the observed minima in the spectra of the BF_3_ molecule originate from the multi-center interference. Note that the spectrum is rotationally symmetric by an angle of $120^{{}^{\circ}}$.

Taking into account rescattering wave packets spreading only from two fluorine atoms (two F atoms whose internuclear radius is parallel to the *x*-axis of the laser field), we obtain the spectrum shown in the upper right panel of Figure 3. In this case, we observe two minima at emission angles $\phi_{f}\approx 50^{{}^{\circ}}$ and $\phi_{f}\approx 290^{{}^{\circ}}$. It is not hard to show that they originate from the two-center destructive interference. The position of both minima can be explained by the two-center rescattering interference formula

(8) $$\left(p_{f}-k_{\mathrm{st}}\right)\cdot R=\left(2m+1\right)\pi ,\;m\in Z,$$

where $p_{f}$ is final electron momentum and $k_{\mathrm{st}}$ is the stationary electron momentum. To obtain the approximate final-momentum value $p_{f}$ (which provides information about the kinetic energy and emission angle) at which the minimum occurs, the appropriate estimated value of $k_{\mathrm{st}}$ must be substituted into the Equation (8). The stationary momentum is determined using the saddle-point method [37]. First, it is necessary to solve the system of the saddle-point equations

(9) $$\begin{array}{ccc} & {\left[k_{\mathrm{st}}+A\left(t_{0}\right)\right]}^{2} & =-2I_{p},\\ & {\left[p_{f}+A\left(t\right)\right]}^{2} & ={\left[k_{\mathrm{st}}+A\left(t\right)\right]}^{2}, \end{array}$$

over $t_{0}$ and *t* to obtain stationary ionization and rescattering times. Solutions of this system of nonlinear algebraic equations are presented in [37] for an ω−2ω bicircular laser field and for the emission angle of (around) 50°. The dominant quantum orbit contribution is obtained for ionization and rescattering times of $t_{0}\approx 0.2T$ and t≈0.64T, respectively. We can now estimate the stationary momentum by $k_{\mathrm{st}}=-\int_{t_{0}}^{t}dt^{\prime \prime }A\left(t^{\prime \prime }\right)/\left(t-t_{0}\right)$ and obtain the final-momentum values, given by Equation (8), and for which the interference minima occur. Plotting these values in the energy-emission angle plane yields the red dotted line in the upper right panel. The dash-dot line that approximately fits the minimum at $\phi_{f}\approx 290^{{}^{\circ}}$ is obtained by inserting the $k_{\mathrm{st}}$ value obtained for $t_{0}\approx 0.86T$ and t≈1.3T, as this is the suitable solution that corresponds to high-energy electrons emitted at $\phi_{f}\approx 290^{{}^{\circ}}$. It is interesting to note that this minimum appears in the same position and with the same shape in HATI spectra for the entire BF_3_ molecule, i.e., when rescattering is calculated with contributions from all four atomic centers. One should notice that the rotational symmetry is broken now.

Considering rescattering from three fluorine atoms (lower left panel) (that lie at the vertices of an equilateral triangle), we conclude that the interference minima are still present but not as sharp as in the case of two atomic interference or the whole molecule. This means that one additional fluorine atom has partially covered the clear interference minima appearing in the two-center case. Because the three fluorine atoms’ positions obey the rotational symmetry by $120^{{}^{\circ}}$, the obtained spectrum is symmetric again with C_3_ symmetry. Finally, in HATI spectra with rescattering contribution from the whole BF_3_ molecule, minima looks like two-center interference minimum calculated for two F atoms, and for minimum at $\phi_{f}\approx 290^{{}^{\circ}}$ it can be fitted with two-center interference minimum calculated for F atoms with internuclear radius R parallel to the *x*-axis of the laser field with $k_{\mathrm{st}}$ value obtained for $t_{0}\approx 0.86T$ and t≈1.3T (red dash-dotted line). The minimum at $\phi_{f}\approx 170^{{}^{\circ}}$ is obtained as a two-center interference minimum, calculated for F atoms with an internuclear radius R that forms an angle of −120° with respect to the *x*-axis of the laser field and with $k_{\mathrm{st}}$ value obtained for $t_{0}\approx 0.53T$ and t≈0.97T (green dash-dotted line). Finally, the black dash-dotted line is obtained from Equation (8) for F atoms with an internuclear radius R that forms a 120° angle with respect to the *x*-axis of the laser field and with $k_{\mathrm{st}}$ value obtained for $t_{0}\approx 0.2T$ and t≈0.64T.

The position of two-center interference minima can be backtracked to molecular orientation and/or internuclear distance of atoms within molecule [47]. To explain how two-center interference minima can account for a more complex four-center interference pattern, we will analyze the contributions of all four atoms in the BF_3_ molecule to HATI spectra in few different ways. The first, more intuitive way, is to consider the four atoms of the BF_3_ molecule as two pairs of atoms and to analyze the two-center interference minima Equation (8) from each pair simultaneously. The only differences between the atomic pairs are the orientation of the vector R and the internuclear distance. In the equilibrium position of BF_3_ the distance between the two F atoms is 2.24 Å, while the distance between the F and B atoms is 1.29 Å, and their internuclear radii are mutually perpendicular (see Figure 1). The resulting two-center interference minimum positions will form two perpendicular lines in the momentum plane. It is clear that analyzing interference pattern from only two atoms provides an incomplete picture of the total interference pattern. However, it is reasonable to expect that the minima in the total interference pattern will appear in the vicinity of the intersection of two complementary two-center interference minima positions.

**Figure 4:**
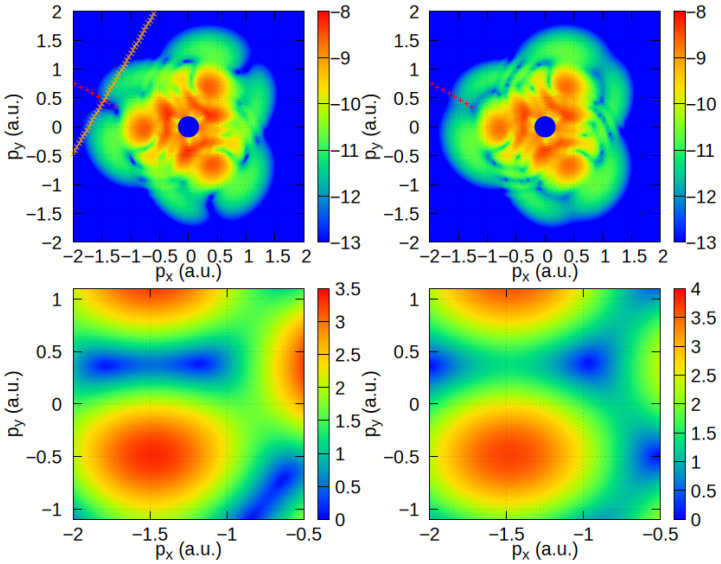
Upper panels: The HATI spectra of the BF_3_ molecule (**left**) and rescattering contributions of the three F atoms (**right**) are presented in momentum plane for the same laser-field parameters as in Figure 2 and $\phi_{m}=90^{{}^{\circ}}$. Red and orange lines represent the corresponding two-center interference minima positions. (Lower panels: Corresponding absolute value of the total interference phase for high-energy (rescattered) electrons and emission angle around 180° presented in momentum plane for the BF_3_ molecule (**left**) and contributions of the three F atoms (**right**).

In the upper left panels of Figure 4, BF_3_ spectrum in the momentum plane is presented. The interference minima in the momentum plane appear shorter but wider, which is more convenient for further discussion. For the brevity and clarity, we only present and discuss the two-center interference minima for the left leaf in characteristic three-leaf symmetric HATI pattern. That is, we consider emission angles around 170°, so $k_{\mathrm{st}}$ in Equation (8) is obtained with $t_{0}\approx 0.53T$ and t≈0.97T as explained above. The spectrum is calculated for a molecular orientation of $\phi_{m}=90^{{}^{\circ}}$ so we first consider contributing atomic pair as two F atoms with internuclear radius that makes an angle of −120° with respect to the *x*-axis of the laser field (see Figure 1). The resulting two-center interference minimum position is presented as red dotted line.

The internuclear radius of the other two atoms (B and F) makes a 210° angle with respect to the *x*-axis of the field, and the corresponding two-center interference minimum position is presented by an orange line. These lines intersect within the area of the deep minimum in the HATI spectrum. Therefore, it is reasonable to expect that the four-center interference pattern has a minimum near this intersection. This explains why the positions of the two-center interference minima in Figure 3 approximately fit the positions of the total four-center interference minima. The upper right panel presents HATI spectrum with rescattering from three F atoms in the momentum plane, and the red dotted line shows the same two-center interference minimum for comparison. We see that the two-center interference minimum is screened by the contribution to the rescattering amplitude from the third F atom.

The other approach, which is more analytical, is to calculate the total four-center rescattering amplitude phase similarly to that in Ref. [33]. Specifically, we calculate the absolute value of the sum over atomic centers, $\sum_{j=1}^{N}e^{iK_{\mathrm{st}}\cdot \rho_{j}\left(\left\{R_{0}\right\}\right)}V_{eK_{\mathrm{st}}}^{j}$, which is part of the transition amplitude in Equation (4). The left lower panel of Figure 4 shows the absolute value of the four-center rescattering amplitude phase for the atomic configuration of the BF_3_ molecule with a molecular orientation of $\phi_{m}=90^{{}^{\circ}}$ and an emission angle of around 180°. A total phase close to zero can be found for the ($p_{x}$, $p_{y}$) coordinates around (−1.5, 0.4) in atomic units. This is the position of the minimum in the corresponding BF_3_ HATI spectra. The right lower panel presents the case of the rescattering amplitude calculated by considering only the three F atoms. There is no deep minimum in the area of interest mentioned above; however, the amplitude is slightly lower than for larger or smaller values of $p_{y}$. This matches the appearance of the corresponding dimmed minima in the HATI spectra obtained with the rescattering amplitude, which was calculated by taking into account the rescattering from the F atoms only (upper right panel).

We use reasonable approximations when considering the absolute value of the multi-center rescattering phase, $|\sum_{j=1}^{N}e^{iK_{\mathrm{st}}\cdot \rho_{j}\left(\left\{R_{0}\right\}\right)}V_{eK_{\mathrm{st}}}^{j}|$, to obtain simple, interpretable results. We approximate the vector $\rho_{j}$ as the position of the center of an individual atom, $r_{j}$. Additionally, we consider $V_{eK_{\mathrm{st}}}^{j}$ to be constant because it does not vary much in the region of interest, i.e., for electron kinetic energies around 9$U_{p}$. The factor affecting the depth of the phase minima is the ratio of its values for the corresponding atoms. For the BF_3_ molecule, we have $V_{eK_{\mathrm{st}}}^{B}/V_{eK_{\mathrm{st}}}^{F}\approx 0.66$. Values of the corresponding ratio close to zero or greater than three for similar molecules result in blurring of these interference minima.

Another simple conclusion concerning the position of the multi-center interference minima in the high-energy part of the HATI spectra follows from the analysis of the above-mentioned multi-center rescattering phase value. Rotating the molecule by 180° around the molecular axis results in the transformation $r_{j}\to -r_{j}$, which leads to the complex conjugate of the sum in the multi-center rescattering phase, which leaves the absolute value unchanged. Since we are considering molecules with C_3_ symmetry, which remain unchanged under a 120° rotation, a 180° rotation is equivalent to a 60° rotation. This can be seen in Figure 2. The position of the minimum in the high-energy spectra for both $\phi_{m}=0^{{}^{\circ}}$ and $\phi_{m}=60^{{}^{\circ}}$ (left panels) is approximately at an emission angle of 70°. The position of the minimum in the high-energy spectra for both $\phi_{m}=30^{{}^{\circ}}$ and $\phi_{m}=90^{{}^{\circ}}$ (right panels) is approximately at an emission angle of 50°.

In calculations concerning minimum positions, we use the most probable, dominant solution for stationary momentum, $k_{\mathrm{st}}$, which corresponds to the dominant quantum orbit. This quantum orbit can only describe the high-energy spectrum for kinetic energies above 8$U_{p}$. For energies below 8$U_{p}$, the contribution of other quantum orbits corresponding to different stationary momentum solutions might not be negligible. The total HATI spectrum is shaped by the properties of the wave function of the molecule, the interference of the emission and rescattering amplitudes from all atomic centers, and by interference of different quantum paths (so called “quantum orbits”). The ionization of considered symmetric molecules in ω−2ω bicircular field that obeys the same C_3_ symmetry, where spatial rotation by 120° is equivalent to translation in time t→t+0.33T, results in complex HATI spectra with the same C_3_ symmetry, which should allow better control in future experiments.

## 4. Conclusions

We extended our theory to present the high-energy electron spectra of polyatomic molecules belonging to the C_3_ symmetry group. We chose the BF_3_ molecule as a representative example. We obtained these spectra using a tailored laser field with the same symmetry as the molecule. Analyzing these spectra provides insight into the internal structure and orientation of these molecules. Due to emission and rescattering occurring at different atomic centers within the molecular target, rich interference patterns can be observed in the molecular spectra. The depth of the interference minima in the high-energy part of the spectra is enhanced by an atom at the center of the molecule but can become blurred if the rescattering potential of the atoms at the center and the vertices differs too much in magnitude. This approach allows us to analyze the internal structures of more complex molecules, particularly those significant in medicine and biology.

## Data Availability

The data presented in this study are available on request from the corresponding author.

## Abbreviations

The following abbreviations are used in this manuscript:

ATI	Above-threshold ionization
HATI	High-order above-threshold ionization
HHG	High-order harmonic generation
LIED	Laser-induced electron diffraction
TDSE	Time-dependent Schrödinger equation
SFA	Strong-field approximation
MSFA	Molecular strong-field approximation
IMSFA	Improved molecular strong-field approximation
HOMO	Highest occupied molecular orbital
GAMESS	General Atomic and Molecular Electronic Structure System

## References

[1] Amini K., Biegert J., Calegari F., Chacón A., Ciappina M.F., Dauphin A., Efimov D.K., Figueira de Morisson Faria C., Giergiel K., Gniewek P. (2019). Symphony on strong field approximation. Rep. Prog. Phys..

[2] Alexander O., Ayuso D., Matthews M., Rego L., Tisch J.W.G., Weaver B., Marangos J.P. (2025). Attosecond physics and technology. Appl. Phys. Lett..

[3] Becker W., Grasbon F., Kopold R., Milošević D.B., Paulus G.G., Walther H. (2002). Above-threshold ionization: From classical features to quantum effects. Adv. At. Mol. Opt. Phys..

[4] Eckart S. (2024). Strong field-induced quantum dynamics in atoms and small molecules. J. Phys. B.

[5] Agostini P., Fabre F., Mainfray G., Petite G., Rahman N.K. (1979). Free-Free Transitions Following Six-Photon Ionization of Xenon Atoms. Phys. Rev. Lett..

[6] Schafer K.J., Yang B., DiMauro L.F., Kulander K.C. (1993). Above threshold ionization beyond the high harmonic cutoff. Phys. Rev. Lett..

[7] Paulus G.G., Nicklich W., Xu H., Lambropoulos P., Walther H. (1994). Plateau in above threshold ionization spectra. Phys. Rev. Lett..

[8] McPherson A., Gibson G., Jara H., Johann U., Luk T.S., McIntyre I.A., Boyer K., Rhodes C.K. (1987). Studies of multiphoton production of vacuum-ultraviolet radiation in the rare gases. J. Opt. Soc. Am. B.

[9] Ferray M., L’Huillier A., Li X.F., Lompre L.A., Mainfray G., Manus C. (1988). Multiple-harmonic conversion of 1064 nm radiation in rare gases. J. Phys. B.

[10] Constant E., Nandi S., Picot C., Prost E., Palakkal S., Lépine F., Loriot V. (2025). High order harmonic generation-based attosecond light sources and applications to quantum phenomena. APL Photonics.

[11] Zhou S., Wang H., Yu D., Xu N., Hu M. (2025). Enhancing High-Order Harmonic Generation Efficiency Through Molecular Size and Orientation Effects: A Pathway to Ultrafast Chemical Dynamics Studies. Molecules.

[12] The Nobel Prize in Physics 2023 Was Awarded to Pierre Agostini, Ferenc Krausz and Anne L’Huillier “for Experimental Methods That Generate Attosecond Pulses of Light for the Study of Electron Dynamics in Matter”. https://www.nobelprize.org/prizes/physics/2023/summary/.

[13] Kfir O., Grychtol P., Turgut E., Knut R., Zusin D., Popmintchev D., Popmintchev T., Nembach H., Shaw J.M., Fleischer A. (2015). Generation of bright phase-matched circularly-polarized extreme ultraviolet high harmonics. Nat. Photonics.

[14] Kjeldsen T.K., Bisgaard C.Z., Madsen L.B., Stapelfeldt H. (2005). Influence of molecular symmetry on strong-field ionization: Studies on ethylene, benzene, fluorobenzene, and chlorofluorobenzene. Phys. Rev. A.

[15] Kumarappan V., Holmegaard L., Martiny C., Madsen C.B., Kjeldsen T.K., Viftrup S.S., Madsen L.B., Stapelfeldt H. (2008). Multiphoton Electron Angular Distributions from Laser-Aligned *CS*_2_ Molecules. Phys. Rev. Lett..

[16] Durá J., Grün A., Bates P.K., Teichmann S.M., Ergler T., Senftleben A., Pflüger T., Schröter C.D., Moshammer R., Ullrich J. (2012). Wavelength Dependence of the Suppressed Ionization of Molecules in Strong Laser Fields. J. Phys. Chem. A.

[17] Lefebvre C., Lu H.Z., Chelkowski S., Bandrauk A.D. (2014). Electron-nuclear dynamics of the one-electron nonlinear polyatomic molecule $H_{3}^{2+}$ in ultrashort intense laser pulses. Phys. Rev. A.

[18] Zhou X.-C., Shi S., Li F., Guo Y.-C., Yang Y.-J., Meng Q.-T., Chen J., Liu X.-J., Wang B. (2020). The interference fringes of above-threshold ionization spectrum of SF_6_ molecules in an IR + XUV laser field. J. Phys. B.

[19] Pullen M.G., Wolter B., Le A.-T., Baudisch M., Hemmer M., Senftleben A., Schröter C.D., Ullrich J., Moshammer R., Lin C.D. (2015). Imaging an aligned polyatomic molecule with laser-induced electron diffraction. Nat. Commun..

[20] Schell F., Bredtmann T., Schulz C.P., Patchkovskii S., Vrakking M.J.J., Mikosch J. (2018). Molecular orbital imprint in laser-driven electron recollision. Sci. Adv..

[21] Chirvi K., Biegert J. (2024). Laser-induced electron diffraction: Imaging of a single gas-phase molecular structure with one of its own electrons. Struct. Dynam..

[22] Mancuso C.A., Hickstein D.D., Dorney K.M., Ellis J.L., Hasović E., Knut R., Grychtol P., Gentry C., Gopalakrishnan M., Zusin D. (2016). Controlling electron-ion rescattering in two-color circularly polarized femtosecond laser fields. Phys. Rev. A.

[23] Beaulieu S., Larroque S., Descamps D., Fabre B., Petit S., Taïeb R., Pons B., Mairesse Y. (2024). Strong-field ionization of chiral molecules with bicircular laser fields: Sub-barrier dynamics, interference, and vortices. Phys. Rev. A.

[24] Hofmann M., Trabert D., Geyer A., Anders N., Kruse J., Rist J., Schmidt L.P.H., Jahnke T., Kunitski M., Schöffler M.S. (2024). Subcycle resolved strong field ionization of chiral molecules and the origin of chiral photoelectron asymmetries. Phys. Rev. Res..

[25] Mosert V., Bauer D. (2016). Photoelectron spectra with Qprop and t-SURFF. Comput. Phys. Commun..

[26] Brown A.C., Armstrong G.S.J., Benda J., Clarke D.D.A., Wragg J., Hamilton K.R., Mašín Z., Gorfinkiel J.D., van der Hart H.W. (2020). RMT: R-matrix with time-dependence. Solving the semi-relativistic, time-dependent Schrödinger equation for general, multielectron atoms and molecules in intense, ultrashort, arbitrarily polarized laser pulses. Comput. Phys. Commun..

[27] Fetić B., Tunja M., Becker W., Milošević D.B. (2022). Extracting photoelectron spectra from the time-dependent wave function. II. Validation of two methods: Projection on plane waves and time-dependent surface flux. Phys. Rev. A.

[28] Winter P., Lein M. (2025). Orientation-dependent ionization rate of diatomic molecules. Phys. Rev. A.

[29] Milošević D.B. (2006). Strong-field approximation for ionization of a diatomic molecule by a strong laser field. Phys. Rev. A.

[30] Hasović E., Milošević D.B. (2012). Strong-field approximation for above-threshold ionization of polyatomic molecules. Phys. Rev. A.

[31] Busuladžić M., Gazibegović-Busuladžić A., Milošević D.B., Becker W. (2008). Angle-Resolved High-Order Above-Threshold Ionization of a Molecule: Sensitive Tool for Molecular Characterization. Phys. Rev. Lett..

[32] Busuladžić M., Hasović E., Becker W., Milošević D.B. (2012). Application of the dressed-bound-state molecular strong-field approximation to above-threshold ionization of heteronuclear molecules: NO vs. CO. J. Chem. Phys..

[33] Hasović E., Milošević D.B. (2014). Strong-field approximation for above-threshold ionization of polyatomic molecules. II. The role of electron rescattering off the molecular centers. Phys. Rev. A.

[34] Habibović D., Jašarević A., Busuladžić M., Milošević D.B. (2024). High-order above-threshold ionisation of diatomic molecules by few-cycle bicircular and orthogonally polarised two-colour pulses. Phys. Chem. Chem. Phys..

[35] Okunishi M., Itaya R., Shimada K., Prümper G., Ueda K., Busuladžić M., Gazibegović-Busuladžić A., Milošević D.B., Becker W. (2008). Two-Source Double-Slit Interference in Angle-Resolved High-Energy Above-Threshold Ionization Spectra of Diatoms. Phys. Rev. Lett..

[36] Quan W., Lai X., Chen Y., Wang C., Hu Z., Liu X., Hao X., Chen J., Hasović E., Busuladžić M. (2013). Resonancelike enhancement in high-order above-threshold ionization of molecules. Phys. Rev. A (R).

[37] Milošević D.B., Becker W. (2016). Improved strong-field approximation and quantum-orbit theory: Application to ionization by a bicircular laser field. Phys. Rev. A.

[38] Atkins P.W., Friedman R.S. (2001). Molecular Quantum Mechanics.

[39] Schmidt M.W., Baldridge K.K., Boatz J.A., Elbert S.T., Gordon M.S., Jensen J.H., Koseki S., Matsunaga N., Nguyen K.A., Su S. (1993). General atomic and molecular electronic structure system. J. Comput. Chem..

[40] Levine I.N. (2000). Quantum Chemistry.

[41] Hasović E., Becker W., Milošević D.B. (2016). Electron rescattering in a bicircular laser field. Opt. Express.

[42] Odžak S., Hasović E., Milošević D.B. (2016). High-order harmonic generation in polyatomic molecules induced by a bicircular laser field. Phys. Rev. A.

[43] Neufeld O. (2025). Degree of Time-Reversal and Dynamical Symmetry Breaking in Electromagnetic Fields and Its Connection to Floquet Engineering. ACS Photonics.

[44] Neufeld O., Tzur M.E., Kfir O., Fleischer A., Cohen O. (2025). Light’s symmetry, asymmetry, and their role in nonlinear optics and ultrafast phenomena. arXiv.

[45] Kahvedžić R., Habibović D., Becker W., Gräfe S., Milošević D.B. (2023). Nondipole Effects in Atomic Ionization Induced by an Intense Counter-Rotating Bicircular Laser Field. Ann. Phys..

[46] Dou Y., Long X., Li P., Ge P., Deng Y., Gong Q., Liu Y. (2025). Controlling nondipole effects with bicircular laser fields. Phys. Rev. A.

[47] Sun R.P., Lai X.Y., Yu S.G., Wang Y.L., Xu S.P., Quan W., Liu X.J. (2019). Tomographic extraction of the internuclear separation based on two-center interference with aligned diatomic molecules. Phys. Rev. Lett..

[48] Maxwell A.S., Figueira de Morisson Faria C. (2020). It is all about phases: Ultrafast holographic photoelectron imaging. Rep. Prog. Phys..

[49] Lai X., Sun R., Yu S., Wang Y., Quan W., Staudte A., Liu X. (2024). Reconstructing Molecular Orbitals with Laser-Induced Electron Tunneling Spectroscopy. Ultrafast Sci..

